# Substance Use-related Emergency Department Visits and Resource Utilization

**DOI:** 10.5811/westjem.2022.1.53834

**Published:** 2022-02-28

**Authors:** Weiwei Beckerleg, Joel Hudgins

**Affiliations:** * The Ottawa Hospital, Division of General Internal Medicine, Ottawa, Ontario, Canada; † University of Ottawa, Faculty of Medicine, Ottawa, Ontario, Canada; ‡ Boston Children’s Hospital, Division of Emergency Medicine, Boston, Massachusetts; § Harvard Medical School, Boston, Massachusetts

## Abstract

**Introduction:**

Substance use-related visits to the emergency department (ED) have been linked to higher service delivery costs, although little is known about the specific services used. Our goal In this study was to describe the recent trends of substance use-related ED visits and assess the association between substance use and specific ED resource utilization.

**Methods:**

We performed a retrospective, cross-sectional study using the National Hospital Ambulatory Medical Care Survey (NHAMCS) data from 2013–2018. All ED visits in the United States for patients ≥18 years of age were included. The primary exposure was having substance use included as a chief complaint or diagnosis, which we identified using the International Classification of Diseases, 9th and 10th revisions, codes. The primary outcome was the use of diagnostic services (including laboratory studies and cardiac monitoring) or imaging studies in the ED.

**Results:**

The study sample included 95,506 visits in the US, extrapolating to over 619 million ED visits nationwide. The total number of ED visits remained stable during the study period, but substance use-related visits increased by 45%, with these visits making up 2.93% of total ED visits in 2013 and 4.25% in 2018. This increase was primarily driven by stimulant-, sedative- (opioids and benzodiazepines), and hallucinogen-related visits. Mental health-related visits rose in parallel by 66% during the same period. Compared to non-substance use-related visits, substance use-related visits were more likely to undergo any diagnostic study (adjusted odds ratio [aOR] 1.28; 95% confidence interval (CI): 1.11–1.47; P = 0.001), toxicology screening (aOR 10.15; 95% CI: 8.84–11.66), but less likely to have imaging studies (aOR 0.62; 95% CI: 0.56–0.68; P <0.0001). In stratified analyses, substance use-related visits with concurrent mental health disorders were more likely to undergo imaging studies (aOR 1.56; 95% CI: 1.09–2.22), while findings were opposite for those without concurrent mental health disorders (aOR 0.64; 95% CI: 0.51–0.71; P for interaction <0.0001).

**Conclusion:**

Substance use- and mental health-related ED visits are rising, and they are associated with increased resource utilization. Further studies are needed to provide more guidance in the approach to acute services in this vulnerable population.

## INTRODUCTION

Substance use is associated with multiple adverse health outcomes, including increased rates of infectious disease, mental health disorders, and mortality.^1^ These outcomes are rapidly increasing over time, with recent data showing that the age-standardized mortality rate due to substance use disorders (SUD) increased by 618.3% between 1980–2014 in the United States.^1^ The most common causes of death associated with substance use were injuries and poisoning, along with other external causes.^2^ Among people ages 15–49 in the US, SUDs and intentional injuries make up close to one third of all deaths.^1^ The poor outcomes associated with substance use, along with its rising prevalence and low treatment rates, create a significant public health issue.^3^ From 2004–2013 the proportion of US adults receiving treatment for SUDs stayed at 1.2–1.3%, representing less than 20% of the population affected.^4^

In light of the low treatment rates, it is not surprising that emergency department (ED) visits related to substance use have risen rapidly.^5^ This increase has created predictable challenges for emergency clinicians and the healthcare system overall, as substance use-related ED visits have been linked to increased length of stay, higher service delivery costs, and higher rates of hospital admissions.^6^–^9^ In addition, increasing ED utilization has outpaced similar increases in hospital inpatient care, meaning the burden of these increased visits has fallen disproportionately on EDs and emergency clinicians.^10^ While resource utilization is high in this population, it remains unclear which specific resources are used in the ED for these visits on a national scale.

Identifying the resource utilization pattern for substance use-related visits could help inform resource allocation and potentially increase standardization of care. This could in turn lead to reduction in unnecessary testing or treatment, and eventually reduce the strain on emergency physicians and the healthcare system overall. With this rationale in mind, we aimed to describe the trends of substance use-related ED visits among US adults nationwide over a five-year period, beginning in 2013, and to evaluate the relationship between substance use and ED resource utilization.

## METHODS

This was a retrospective, cross-sectional study using data from the National Hospital Ambulatory Medical Care Survey (NHAMCS), which is conducted by the National Center for Health Statistics (NCHS).^11^ We included data from January 1, 2013–December 31, 2018. The NHAMCS is an annual, national probability sample of ambulatory care visits throughout the US and collects data on visits to hospital-based EDs. The survey employs a four-stage probability design with samples of area primary sampling units (PSU, hospitals within PSUs, clinics within outpatient departments, and patient visits within emergency service areas (ESA). Within each ESA, patient visits were systematically selected over a randomly assigned four-week reporting period. There were approximately 2000 PSUs that covered 50 states and the District of Columbia, and approximately 600 hospitals. Data collection was overseen by the US Bureau of the Census, which provided field training on data abstraction for participating hospital staff.

Ethics approval was obtained from the research ethics board at our home institution.

**Population Health Research Capsule**

What do we already know about this issue?

*Substance use-related visits to the emergency department (ED) have been increasing and are linked to higher service delivery costs.*

What was the research question?

*We aimed to assess the association between substance use and specific ED resource utilization.*

What was the major finding of the study?

*Patients with substance use-related ED visits are more likely to undergo diagnostic tests, including toxicology screening.*

How does this improve population health?

*Results from this study support the need for future studies to provide guidance in the approach to acute services for substance use-related ED visits.*

### Study Population

All ED visits for patients ≥18 years of age were included. We excluded visits to the ED made by patients younger than 18, visits for which the chief complaint or diagnoses were missing, and visits with missing data on use of diagnostic services, medications, procedures, disposition decision, or use of mental health consultation services.

### Exposures and Covariates

The primary exposure was defined as having substance use listed as a chief complaint or diagnosis in the visit, as identified by the *International Classification of Diseases* 9^th^ and 10^th^ revisions (ICD) codes. The ICD codes were taken from previously published briefs by the Health Care Utilization Project.^5^, ^12^ Substances of interest included alcohol (ethanol), opioids, cannabis, cocaine, amphetamines, hallucinogens, and other recreational substances of abuse that affect the central nervous system. Substances were further broken down into five categories as defined by previous literature: 1) alcohol; 2) opioid, sedative/hypnotic, or anxiolytic; 3) cocaine, amphetamine, psychostimulant, or sympathomimetic; 4) cannabis or hallucinogen; and 5) other/unspecified or combined.^7^ The reference group consisted of ED visits without substance use as a diagnosis or chief complaint.

Covariates of interest were defined a priori and identified from literature review.^6^–^8^ They included age, gender, ethnicity, homelessness, burden of comorbidities, presence of mental health disorder, geographical region, metropolitan statistical area, payment source, day of visit, and arrival time. Mental health disorder was treated as a separate diagnosis from SUD to specifically examine the trend of substance use-related visits and to emulate previous studies in this area.

### Outcomes

The primary outcomes of interest consisted of the use of any diagnostic services, toxicology screens or imaging studies in the ED. Diagnostic services included laboratory investigations, toxicology screens, imaging studies, electrocardiograms, and cardiac monitoring. Imaging studies included all imaging carried out in the ED, such as radiographs, ultrasounds, computed tomography (CT), and magnetic resonance imaging. Secondary outcomes consisted of number of procedures performed (eg, intravenous fluids, casts, intubation, lumbar puncture, etc), number of medications administered, disposition, and use of mental health consultation services in the ED. These variables were identified using pre-existing matching labels in the NHAMCS database.^11^

### Statistical analysis

The NHAMCS used a multistage estimation procedure to produce essentially unbiased estimates. The first step included inflation by reciprocals of selection probabilities, which was the product of the probability at each sampling stage. The second step adjusted for survey nonresponse, which included inflating weights of visits to hospitals or EDs similar to nonrespondent units, depending on the pattern of missingness. During data analysis, survey procedures were used (using the svy command) and patient visit weights were applied to obtain the total estimated ED visits from sampled visits (using the PATWT variable). As per the NHCS, sampled visits with relative standard error of 30% or more and observations that were based on fewer than 30 sampling records may yield unstable estimates. These were specifically indicated and later excluded from analysis.

We performed univariate analysis using chi-squared test to assess the association between substance use and each of the categorical covariates. To test for linear trend in substance use-related visits over time, we applied a logistic regression model with substance use as the dependent variable and time (measured in years) as the independent variable. Univariate and multivariable logistic regression were used to assess the unadjusted and adjusted associations between substance use and each of the outcomes, respectively. All listed covariates, with the exception of mental health disorder, were included in the multivariable model. We reported odds ratios for all logistic regression analyses, along with 95% confidence intervals. For the primary and secondary outcomes of interest, *P*-value for significance was determined to be 0.005 after applying Bonferroni correction, to minimize family-wise error rate in the setting of multiple comparisons. To evaluate mental health disorder as a potential effect modifier, we assessed the relationship between substance use and primary outcomes using a stratified analysis. The *P*-value for interaction was obtained from a multivariable logistic regression model. Missing data were handled using complete case analysis, given that the percentage of missingness was small, and complete data were available for both the exposures and outcomes. All data analyses were carried out using STATA version 15 (StataCorp LLC, College Station, TX).

## RESULTS

**Figure 1:**
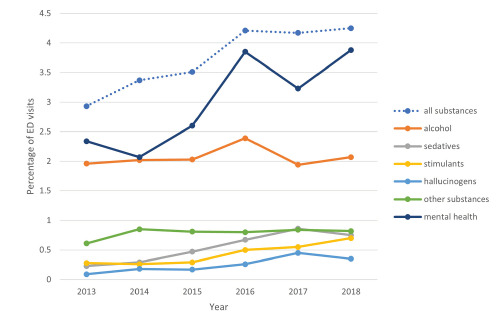
Temporal trend of substance use- and mental health-related emergency department visits over time. *ED*, emergency department.

From 2013–2018, substance use-related ED visits increased from 2.926 to 4.132 million visits, or from 2.93% to 4.25% of total ED visits during the same period, which translates to a 45% relative increase. Non-substance use-related ED visits (reference group) remained stable during the same period, with 93.17 million visits in 2018 compared to 96.98 million visits in 2013. The rise in substance use-related ED visits was driven by sedatives, stimulants, and hallucinogens, with alcohol and other substance use-related visits being relatively stable (Figure 1). There was a parallel increase in mental health-related visits, with these visits making up 2.34% of total ED visits in 2013 and 3.88% in 2018, representing a 66% relative increase.

Among substance-use related visits, the 25–44 age group made up 44.58% of visits, as compared to 35.49% of the non-substance related group (*P* <0.0001). There was also a male predominance among substance use-related visits: males accounted for 63.38% of visits in the substance group vs 41.74% in the reference group (*P* <0.0001). While the West geographic area accounted for only 21.34% of all ED visits, it made up 29.67% of substance use-related visits. In addition, substance use-related visits were much more likely to happen during the night shift (11 pm – 7 am), with 27.07% of all substance use-related visits taking place then compared to 14.81% in the reference group (*P* <0.0001) (Table 1). Mental health issues were more prevalent in the substance use group compared to the reference group, present in 14.48% vs 2.99%, respectively.

With regard to the primary outcomes, patients associated with substance use-related visits were more likely to undergo any diagnostic study (adjusted odds ratio [aOR] 1.28; 95% CI: 1.11–1.47, *P* = 0.001) and toxicology screening (aOR 10.15; 95% CI: 8.84–11.66; *P* <0.0001); however, they were less likely to have imaging studies (aOR 0.62; 95% CI: 0.56–0.68; *P* <0.0001) (Table 2).

There were no significant differences in the use of medications or procedures between the substance use and reference groups, with the differences in means being 0.08 (95% CI: −0.06–0.21; *P* = 0.28) and 0.04 (95% CI: 0.01–0.07; *P* = 0.02), respectively (Table 3). Substance use-related visits were associated with higher odds of admission or transfer to another facility (aOR 1.73; 95% CI: 1.53–1.96; *P* <0.0001) and higher odds of receiving a mental health consult [aOR 5.70; 95% CI: 4.47–7.28; *P* <0.0001).

With regard to stratified analyses those patients with mental health disorders were more likely to have imaging studies, and this reached statistical significance for interaction (*P* <0.0001). For substance use-related visits without the concurrent presence of a mental health disorder, the aOR of undergoing any imaging study was 0.65 (95% CI: 0.58–0.72), and for substance use-related visits with concurrent mental health disorder, the aOR of undergoing any imaging study was 1.44 (95% CI: 1.03–2.00). All substance use-related ED visits were more likely to undergo toxicology screening, but those without concurrent mental health disorders were even more likely to receive screening, with aOR of 11.47 (95% CI: 9.87–13.35). The presence of a mental health disorder did not have an impact on the relationship between undergoing any diagnostic study in ED and substance use (Table 4).

## DISCUSSION

Consistent with previously published work, our study shows that sedative-, stimulant-, and hallucinogen-related ED visits continue to increase rapidly compared to alcohol and other substances of abuse.^6^,^13^,^14^ Substance use-related ED visits are more likely to result in diagnostic investigations overall, admission or transfer to another facility, and mental health consultations. Conversely, they are less likely to result in imaging studies. While the higher rate of admission/transfer and mental health consultations for substance use-related ED visits has been reported previously,^7^,^15^ to our knowledge the use of diagnostic services has not yet been assessed at the national level.

Among the common substances of abuse, the rapid increase in stimulant-related ED visits in recent years is remarkable; in 2018, the percentage of stimulant-related visits matched that of sedative-related visits (including opioid, benzodiazepines, and other sedatives), representing approximately 0.7% of total ED visits. This is consistent with other study findings that have reported a rise in prevalence of stimulant use across all age groups from 2010–2014, with adults between 20–64 years the most affected.^16^ Our study also showed that the rise in stimulant-related visits was more pronounced in the 18–44 age group (OR 1.28), compared to the > 45 years age group (OR 1.13). The most frequently cited motivation for stimulant use among adults was performance enhancement,^17^ which supports the need to improve public education for young adults on the addictive potential of stimulants and restricting prescriptions to appropriate clinical indications only.

Regarding the use of diagnostic services in the ED for substance use-related visits, research has been relatively sparse. Our study showed that substance use-related visits are more likely to receive diagnostic services overall (including both laboratory and imaging studies) and toxicology screening. Some studies have called into question the routine practice of ordering urine drug screens for substance-related visits and laboratory studies in general for mental health-related visits, as they have rarely led to changes in management.^18^,^19^ The American Psychiatric Association (APA) and the American College of Emergency Physicians (ACEP) both support targeted diagnostic investigations for patients presenting with acute psychiatric symptoms, instead of routine testing.^20^,^21^ However, drug testing is often required as part of initial assessment to enter treatment facilities, regardless of medical indication or emergency healthcare team preferences.^22^ Although most of the studies on this topic focused on mental health-related ED visits, the often-overlapping presentations of substance- and mental health-related visits argue for standardization of practices to diagnostic services.

In terms of the use of imaging studies specifically, both ACEP and the APA support individual assessment of risk factors to guide brain imaging in the ED for mental health-related visits, due to low yield of routine imaging.^20^,^21^ There are no recommendations made regarding substance use-related visits given limited evidence. In contrast to our finding of substance use-related visits being associated with less use of imaging studies, previous work has shown a rising trend in the use of CT along with the rise of opioid-related visits.^6^ However, that study did not assess the use of CT in relation to a non-substance use reference group and did not include other imaging modalities. The lower rate of utilization of imaging studies could be explained by the possibility that imaging was not needed for management or disposition after completion of laboratory screening in substance use-related visits. In addition, since substance use-related visits occurred disproportionately after hours, imaging might not be readily available after hours in smaller centers. Visiting hours were adjusted for as a potential confounder; so the latter explanation is considered less likely.

Notably, the presence of a mental health disorder made it more likely for patients with a substance use diagnosis to undergo imaging studies. It is well documented that patients with serious mental health disorders have higher mortality rates than those without, attributable to both injuries and chronic diseases.^2^ It is, therefore, possible that additional imaging studies were needed because of increased medical complexity. Furthermore, the presence of SUDs was associated with significantly increased rates of mental health consultations in the ED, which in turn have been shown to be associated with increased ED length of stay.^24^ These findings support the fact that healthcare is more costly for patients with mental health or SUDs, highlighting the need to address physical and mental health in an integrated fashion.^23^ In fact, multiple studies have shown the effectiveness of case coordination and combined medical and behavioral health clinics to help decrease substance use- or mental health-related ED visits.^25^,^26^

## LIMITATIONS

Our study results should be interpreted in the context of several limitations. First, only associations and no causal relationships could be made due to the cross-sectional nature of the study. Second, it is possible that some substance use-related ED visits represented repeated visits over time, meaning the statistical methods used in the analysis could yield biased results away from the null. As the NHAMCS is an event-level database, it is not possible to ascertain this as data linkage could not be performed. Third, the study results relied heavily on ED reporting and ICD codes, which could be subject to inaccuracies and bias the results toward the null, although steps were taken to mitigate this through staff training.

Fourth, due to limitations in sample size, detailed analysis on the specific types of diagnostic services or imaging modalities, with the exception of toxicology screening, were not done. Further studies incorporating data from previous years would be needed to obtain more granular data. Fifth, due to concerns about multiplicity, resource utilization pattern with respect to the subgroups of substances analyzed can only be used for hypothesis-generating purposes. Furthermore, improved screening strategies for substance use in the ED could have contributed to the increase in visits, following the emergence of evidence demonstrating improved outcomes associated with ED-initiated interventions, biasing the results away from the null.^27^ Finally, this study did not include information on ED-initiated substance use treatment or outpatient referral pattern over time, making it difficult to comment on specific strategies to help improve care for patients with SUD in the ED. In summary, many of the limitations arose from the design of the survey itself and were difficult to mitigate at the data analysis stage.

## CONCLUSION

Substance use- and mental health-related ED visits are rising and are associated with increased resource utilization. Increasing mental health support will continue to be needed in the ED, along with support for ED clinicians in the management of common substances of abuse, especially sedatives and stimulants. Additional studies are needed to understand the pattern of resource utilization in the ED for substance use- and mental health-related visits, and to assess the optimal approach to acute care management for these visits.

## Figures and Tables

**Table 1:** Descriptive table of baseline demographic characteristics of study population by exposure categories.

	All visits (%/N) N = 95,506	Substance use (%/N) N = 4,050	Non-substance use (%/N)* N = 91,456
Age			
18–24	14.30 (13,386)	14.49 (552)	14.29 (12,834)
25–44	35.83 (34,561)	44.58 (1,787)	35.49 (32,774)
45–64	29.00 (27,974)	34.38 (1,467)	28.79 (26,507)
65–74	9.55 (8,952)	5.25 (193)	9.72 (8,759)
≥75	11.31(10,633)	1.31 (51)	11.70 (10,582)
Gender			
Male	42.55 (41,253)	63.38 (2,680)	41.74 (38,573)
Female	57.45 (54,253)	36.62 (1,370)	58.26 (52,883)
Ethnicity			
Non-Hispanic White	61.05 (57,890)	62.43 (2,402)	60.99 (55,488)
Non-Hispanic Black	23.12 (21,871)	19.98 (945)	23.24 (20,926)
Hispanic	13.02 (12,572)	14.18 (554)	12.98 (12,018)
Non-Hispanic other	2.82 (3,173)	3.41 (149)	2.79 (3,024)
Residence			
Homeless / shelter	0.89 (1,355)	6.65 (398)	0.66 (957)
Private residence / nursing home	95.13 (90,338)	86.46 (3,382)	95.47 (86,956)
Other	1.52 (1,466)	3.19 (121)	1.45 (1,345)
Missing	2.46 (2,347)	3.70 (149)	2.42 (2,198)
Mental health disorder			
Present	2.99 (3,513)	14.48 (602)	2.55 (2,911)
Absent	97.01 (91,993)	85.52 (3,448)	97.45 (88,545)
Number of chronic conditions (mean/SE)	1.09(0.03)	1.94 (0.05)	1.06 (0.03)
Payment source			
Private insurance	26.84 (26,149)	18.90 (744)	27.15 (25,405)
Public insurance	49.72 (47,971)	48.83 (2,046)	49.76 (45,925)
Self-pay	11.31 (10,295)	15.73 (609)	11.14 (9,686)
Other	2.68 (2,697)	3.17 (167)	2.66 (2,512)
Missing	9.45 (8,412)	13.36 (484)	9.29 (7,928)
Geographic region			
Northeast	16.04 (17,938)	18.35 (945)	15.95 (16,993)
Midwest	24.33 (23,022)	23.53 (790)	24.36 (22,232)
South	38.29 (33,625)	28.46 (1,068)	38.67 (32,557)
West	21.34 (20,921)	29.67(1,247)	21.01 (19,674)
Metropolitan statistical area status (MSA)			
MSA	83.46 (81,054)	89.76 (3,734)	83.22 (77,320)
Non-MSA	16.64 (14,452)	10.24 (316)	16.78 (14,136)
Day of week			
Weekday	73.44 (70,225)	70.21 (2,853)	73.57 (67,372)
Weekend	26.56 (25,281)	29.79 (1,197)	26.43 (24,084)
Arrival time			
7 AM – 2:59 PM	42.32 (39,981)	29.37 (1,212)	42.82 (38,769)
3 PM – 10:59 PM	41.04 (38,946)	42.26 (1,675)	40.99 (37,281)
11 PM – 6:59 AM	15.27 (14,575)	27.07 (1,055)	14.81 (13,520)
Missing	1.37 (1,994)	1.30 (108)	1.37 (1,886)

* Numbers represent the actual number of observations and percentages obtained after applying sampling procedures to account for complex sampling design.

**Table 2:** Logistic regression models predicting any diagnostic test and any imaging performed in the emergency department.

	Any substance use ***
Any diagnostic study (unadjusted OR and 95% CI)*	1.17 (1.02–1.34)
Any diagnostic study (adjusted OR and 95% CI)**	1.28 (1.11–1.47)
Toxicology screen (unadjusted OR and 95% CI)	14.45 (12.82–16.30)
Toxicology screen (adjusted OR and 95% CI)	10.15 (8.84–11.66)
Any imaging (unadjusted OR and 95% CI)	0.58 (0.53–0.64)
Any imaging (adjusted OR and 95% CI)	0.62 (0.56–0.68)

* Reference group consists of visits without substance use as a diagnosis.

** Adjusted variables include age, gender, race, number of chronic conditions, region, metropolitan statistical area, payment method, residence, arrival time, and day of the week.

*** Complete case analysis was used to handle missing data. N = 95,506 for all logistic regression analyses performed.

Any diagnostic study includes laboratory investigations, radiology services, and others such as cardiac monitoring. Any imaging includes all radiology services such as radiographs, computed tomography, magnetic resonance imaging, and ultrasound.

*OR*, odds ratio; *CI*, confidence interval.

**Table 3:** Regression models predicting use of medications and procedures, disposition, and mental health consultations in the emergency department.

	Medications* (mean, 95% CI)**	Procedures* (mean, 95% CI)**	Admission/transfer (OR)**	Mental health consult
Any substance use	0.08 (−0.06–0.21)	0.04 (0.01–0.07)	1.73 (1.53–1.96)	5.70 (4.47–7.28)
Alcohol use	-	-	1.28 (1.07–1.55)	3.91 (2.87–5.34)
Sedative use	-	-	2.31 (1.80–2.97)	3.81 (2.55–5.69)
Stimulant use	-	-	2.20 (1.64–2.95)	6.93 (4.53–10.60)
Hallucinogen use	-	-	2.62 (1.52–4.52)	4.20 (2.34–7.54)
Other substance use	-	-	2.40 (1.78–3.24)	5.60 (4.00–7.84)

* Mean value represents the difference in the mean between visits with substance use and those without.

** Means and odds ratios were adjusted for gender, age, race, total number of chronic conditions, payment method, residence, region, metropolitan statistical area, day of the week, and arrival time.

Reference group consists of visits without substance use as a diagnosis.

*OR*, odds ratio; *CI*, confidence interval.

**Table 4:** Subgroup analysis for primary outcomes by presence of mental health disorder.

	Presence of mental health disorder	Absence of mental health disorder	*P*-value *
Any diagnostic test (OR and 95% CI)	1.04 (0.76–1.43)	1.33 (1.15–1.53)	0.14
Toxicology screen (OR and 95% CI)	2.23 (1.68–2.97)	11.47 (9.87–13.35)	<0.0001
Any imaging (OR and 95% CI)	1.44 (1.03–2.00)	0.65 (0.58–0.72)	<0.0001

* *P*-values for interaction obtained from adjusted Wald test.

*OR*, odds ratio; *CI*, confidence interval.
